# Supplementary figures and images for: Footfall patterns and stride parameters of Common hippopotamus (Hippopotamus amphibius) on land
Source: PeerJ. 2024 Jul 3;12:e17675. doi: 10.7717/peerj.17675 (PMC11227274; doi:10.7717/peerj.17675)

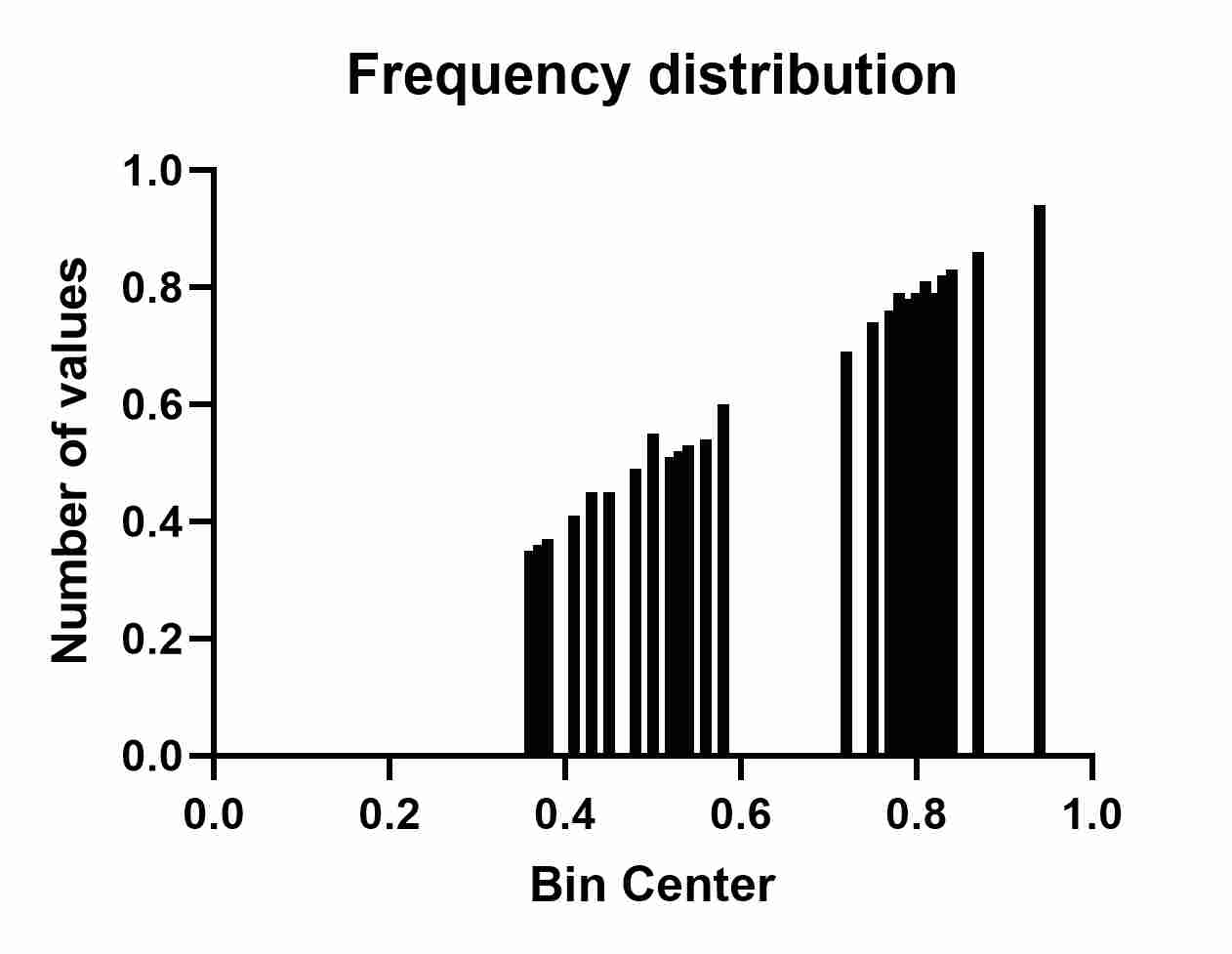

Supplement: Supplemental Information 2 — Each trial’s mean DF was automatically binned in order to visualise the frequency distribution of these data across all 46 trials. The two main "running" (low DF and "bin center") and "walking" (high DF and "bin center") clusters are evident. [file peerj-12-17675-s002.jpg]
